# Is the qualitative research interview an acceptable medium for research with palliative care patients and carers?

**DOI:** 10.1186/1472-6939-9-7

**Published:** 2008-04-24

**Authors:** Marjolein Gysels, Cathy Shipman, Irene J Higginson

**Affiliations:** 1 Palliative Care, Policy & Rehabilitation, School of Medicine, King's College London, London, SE5 9RJ, UK; 2 Barcelona Centre for International Health Research (CRESIB), Barcelona, Spain

## Abstract

**Background:**

Contradictory evidence exists about the emotional burden of participating in qualitative research for palliative care patients and carers and this raises questions about whether this type of research is ethically justified in a vulnerable population. This study aimed to investigate palliative care patients' and carers' perceptions of the benefits and problems associated with open interviews and to understand what causes distress and what is helpful about participation in a research interview.

**Methods:**

A descriptive qualitative study. The data were collected in the context of two studies exploring the experiences of care of palliative care patients and carers. The interviews ended with questions about patients' and carers' thoughts on participating in the studies and whether this had been a distressing or helpful event. We used a qualitative descriptive analysis strategy generated from the interviews and the observational and interactional data obtained in the course of the study.

**Results:**

The interviews were considered helpful: sharing problems was therapeutic and being able to contribute to research was empowering. However, thinking about the future was reported to be the most challenging. Consent forms were sometimes read with apprehension and being physically unable to sign was experienced as upsetting. Interviewing patients and carers separately was sometimes difficult and not always possible.

**Conclusion:**

The open interview enables the perspectives of patients and carers to be heard, unfettered from the structure of closed questions. It also enables those patients or carers to take part who would be unable to participate in other study designs. The context is at least as important as the format of the research interview taking into account the relational circumstances with carers and appropriate ways of obtaining informed consent. Retrospective consent could be a solution to enhancing participants control over the interview.

## Background

The user perspective is increasingly seen as important in evaluations of health care [1]. This requires interviewing seriously ill patients. Qualitative research is consistent with the philosophy of palliative care – promoting care for the whole person through a multidisciplinary approach. However, there are serious ethical considerations involved and qualitative research should be submitted to rigorous criteria [2,3]. The concerns expressed regarding qualitative research centre on its merit and its ethical implications. Assumptions exist, which come from a narrow biomedical perspective, that research which does not start from hypotheses, or does not produce one definitive result, or is not generalisable, does not have value and is therefore not appropriate [4,5]. Challenges of qualitative research are also found in the personal or relational nature of narrative or in-depth interviewing [6].

These concerns are contradicted by evidence documenting the contribution of qualitative research to care at the end-of-life [7-9], including the seminal work by Glaser and Strauss [10]. Qualitative research can address meaning-centred questions that are not easily quantifiable, especially relevant in a complex area. A growing body of research, especially from the bereavement and counselling work has shown that qualitative research is more likely to have beneficial effects than to cause distress [11-13]. Well-conducted interviews by experienced researchers may have counselling qualities [6].

Due to a lack of familiarity with qualitative research, judgements by ethics committees are often based on criteria designed to evaluate experimental research [14] and therefore treated unfairly [15-17]. Decisions are driven by a priori assumptions of vulnerability rather than on evidence of what is actually harmful. A gap exists between prescriptive theoretical approaches and the practicalities of conducting the actual study [18]. Therefore, we need empirical data in order to plan research which is appropriate and acceptable [19], and to support ethics committees in making adequate decisions [20]. This entails a knowledge-base of the methods that cause least harm [21].

This study investigated palliative care patients' and carers' perceptions of the benefits and problems associated with open interviews and to understand what causes distress and what is helpful about participation in a research interview.

## Methods

The data were collected in the context of two qualitative studies exploring the experiences of involvement in research of palliative care patients and their carers. One study (Experience of breathlessness study or EBS) took a comparative approach towards patients suffering from breathlessness, with different conditions. The other study (Experience of cancer study or ECS) focused on cancer patients. We combined the samples of the two studies as similar issues emerged regarding recruitment and interviewing among the patients with cancer. Both studies took place in a teaching hospital and were concurrent (between July 2005 and March 2006).

The sample was purposive (see Additional file 1). Demographic and clinical information about patients was retrieved from patients' records or letters.

We used semi-structured, open-ended interviews. These were exploratory, allowing respondents to touch on any topic relevant to them, but a topic guide ensured that all necessary topics were covered. The interviews ended with questions about participants' thoughts on the studies and whether this had been a distressing or helpful event. This was part of debriefing after the interview. The interviews lasted between 40 and 150 minutes, all were tape-recorded and most transcribed verbatim, (in two instances the recorder broke down, but the interviews were reconstructed immediately after). A notebook was used to record field notes.

We used a qualitative descriptive analysis. The representations are generated from the answers to the questions in the interviews and the observational and interactional data obtained in the course of the study.

The study had obtained ethics approval by the Local Research Ethics Committee and the relevant R&D committees and the community of Lambeth and Southwark. A patient and carer coding system ensured confidentiality throughout the study.

## Results

In total we conducted interviews with 104 participants. In EBS, with 56 patients, and 25 carers. In ECS we conducted interviews with 23 participants, 20 patients and 3 carers (see Additional file 1).

### The format of the interview

#### Is it helpful or does it cause distress?

Most participants responded positively to the interviews. Comments were received on the quality and conduct of the interview, showing the importance of an empathic researcher, interested but not-judgmental. Others appreciated the format of the interview to allow for meaningful narration. Sharing thoughts was experienced as therapeutic.

(6) "It's been so good to tell you all this, you need it from time to time."

It offered an occasion to vent frustrations which could result in true life stories. After the interview patients often expressed their thanks for having been able to make sense of their experiences. The benefit of the medium of the research interview was not only limited to a personal level. Patients and carers felt they were asked to contribute to research with the purpose of improving services, which meant that their views mattered and this had an empowering effect.

(3) "Of course doctors know more about every little thing that is going on in your body, but we are living with it and we need to tell what it is like..."

(23)"...but I've always said listen (to patients) and also in paediatrics, listen to mothers because they know their children more than anybody else."

Only a few participants (4/104) expressed doubts about the helpfulness of the interview:

(7)"I don't know if this is of any help, my personal experiences, maybe somewhere along the line."

(42)"Maybe (it is helpful) for someone else, not for me."

One out of 104 participants reacted negatively to the question if the interview had in any sense been helpful to him:

(45)"This is a really silly question! I am answering all these questions and then you ask whether this has been helpful to ME."

Nobody said that the interview had caused distress. Some patients qualified this by admitting that the topic of the future had been the most challenging. We had been very cautious about this topic and asked participants first if they wouldn't mind speaking about it. One carer present at an interview with the patient had asked the researcher to skip the section, she said: "I will have to pick up the pieces afterwards". Where talking about the future was possible, it facilitated thoughts about preferences for end-of-life care, and this was considered helpful.

(41)"It's been interesting and you've made us think, with an open mind."

The interviews with carers separately evoked emotional reactions. Almost all the interviews led to tears. Sometimes the interview had to be stopped allowing the carer some time to recover. When the researcher checked if they were all right, they all wished to continue the interview. These carers declared afterwards that they did not find the interview distressing.

(40)"It wasn't distressing. [I] think it's quite useful to talk things through. I know I got upset but that's just the way I am. It wasn't the interview that upset me, it's just me, no it was fine."

### Contextual factors of the interview

#### Informed consent

Patients and carers did not provide informed consent in the manner anticipated by ethics committees as a simple one-off statement and straightforward proof of a person's willingness to participate. We found that these forms were often too long and needed discussion, especially with patients in the advanced stages of illness. This reduced the time available for the actual interview. Interviews with the frailer patients had to be stopped long before all the topics were covered because they became too tired.

The consent forms often caused suspicion. Patients' willingness to take part in the interview was a separate issue to concerns about the forms. When presenting the forms a couple of patients exclaimed: "I am not going to sign anything!", then they started telling about their illness and the care they received. Interrupting these patients and asking their attention to sign the form first, would have been impolite and disrespectful to the person who was confiding their story to the researcher. This led her to present the consent forms after the interview, which allowed for rapport and confidence building during the interview. Although the suspicion about signing papers was not entirely gone when asked again, they eventually gave written consent when looking back at the interview.

In EBS, written consent was sometimes problematic, where disability was such that it required considerable effort from the patient to sign the forms. In some cases this was experienced as upsetting, emphasising one's level of disability. In one case, a patient who had not accepted his situation did not accept his wife's offer to sign as a proxy, but insisted on signing the form himself, which was a painful start to the interview. For a lady who was blind, written consent did not pose a problem at all. She suggested the researcher read out the form and direct her hand to the place to sign. These incidents show that consent is something which is negotiated in a relationship between the patient or carer and the researcher.

#### Interviewing patients and carers jointly or separately?

Initially, when we asked patients and carers to be interviewed we intended to speak separately with them. We wanted to know about their needs for care, how the illness impacted on their life and relationships, and their attitudes and provisions for the future. From the literature we know that these are sensitive issues which are not always openly discussed between partners who often protect each other.

We did not always succeed to conduct separate interviews, except for the interviews with MND patients (see Additional file 1). The wish to be present at one another's interview came from both the carer and the patient. Often the more vulnerable patients relied strongly on the input of the carer. Joint interviews were conducted sometimes because there was a lack of space in the house and it was not appropriate to send the carer outside. We did not insist on separate interviews as couples were experiencing the consequences of an illness very much together and were interested in what the other party was telling. In some cases the interview wouldn't have been so interesting if they would have been separate, couples told a joint story, prompting each other and building on each others comments. This could also lead to difficulties, for example when one person would broach an issue which the other had not wanted to disclose or when something was said that had never been expressed so explicitly before.

(3)Carer: "I realise as from Tuesday when they told me that, technically, the hospital has given up on him, that a different department would be taking over."

Patient: "They didn't actually say they'd given up."

Carer: "No I mean, that's why I said in inverted commas."

Where possible, interviews with carers were arranged separately at a later date. These could produce completely different information. When carers eventually thought about themselves and their role as carer they imparted issues they sometimes had never talked about before. A different view surfaced with uncertainties, admitting the burden of caring, and reflecting on one's limits.

## Discussion

Most of the participants evaluated the interview positively and nobody said it had caused distress. The interviews were found to be therapeutic and empowering. The informal nature of the interview allows for an empathic relationship between the researcher and participant which is appreciated. The flexibility of an open interview allows the researcher to follow the order of topics that participants talk about and how they structure these into an explanation that makes sense to them. They can adjust to patients' levels of understanding, reformulating questions into more familiar language, or announcing sensitive topics and checking their appropriateness for discussion. The open interview has the added benefit of being able to reach those patients or carers who are unable to participate in other study designs and who are therefore usually missed by research.

One of the challenges of palliative care is its focus on both patients and those close to them. Patients and carers may have vastly different needs. Conflict can exist between the patient and carer about whether or not to participate in research and this can lead to gatekeeping by one of the parties which presents dilemmas for researchers. The approach in research is mostly to conduct separate interviews with each so that the genuine patient or carer view can surface. It avoids distress for the carer to have to listen to patients' concerns and for the patient to be reminded of the carer's burden. However, we found that interviewing patients and carers separately was not always possible and therefore, we interviewed them as dyads when they wished so. Davies et al. [22] reported a similar finding that the carer was unwilling in some cases to leave the patient when interviewed. In the light of their research question focusing on awareness of prognosis they argued for the value of separate interviews. Here, however, we recognise that separate or joint interviews can produce completely different but both valuable data, giving insight in either the specific patient's or carer's perspective independent from the other party's perspective, or the experiences constructed together as couples both affected by advanced illness. Their value is therefore dependent on the aims of the study and whether or to what extent these data answer what the study is trying to find out. We need to ensure that we are aware of the different data these different approaches can produce and that mixing them could cause flawed results.

It is important for future studies to anticipate these issues. Recruitment strategies and questions of whether to approach patients and carers jointly or separately and if so, who first, need to be thought through. Also, different interview strategies could be considered. It may be appropriate, for example, to undertake an initial joint interview and then interview the carer separately in a different location. Another solution could be to conduct simultaneous separate interviews by two researchers, however, this has resource implications.

Informed consent is important to enable patients and carers to be fully aware of what they are participating in. However, formal procedures sometimes interfered with the flexible and caring approach necessary to communicate with severely ill patients. Consent forms were sometimes read with apprehension and appeared too long for patients with low energy levels. Conditions for informed consent may need to be reconsidered for qualitative research in palliative care, not given as a one-off proof of a person's willingness to participate, but maintained in an ongoing process [23]. Different criteria for judging the value and ethical implications of qualitative studies in palliative care are needed. Tight requirements designed for interventional research may be causing excessive effort or distress for the people which they intend to protect from harm. In some cases, the consent forms signalled to patients a relinquishment of their rights. We found that retrospective consent was one of the ways of operationalising consent in a vulnerable population with an in-built suspicion towards signing forms. We used retrospective consent as part of a process approach; first we explained the purpose of consent to patients, presented the forms and if there was any doubt we would delay the decision. People would participate spontaneously in an informal open interview, willingly answering the researcher's questions without being deterred by the formality of the consent procedures. Having completed the interview the participants did not object to signing the consent form as they better understood what had been said during the interview and could judge whether they found their contribution valuable.

The data could be challenged with the objection that part of the feedback we received on patients' and carers' responses to the interview was motivated by social acceptability. However, the qualitative methodology made it possible to check the intentions through which these responses were driven. The acceptability of the interviews was confirmed by observations, and the contextual information obtained by interaction with participants. We verified the qualitative information gained by looking at the internal consistency of the interviews. This triangulatory approach makes us confident that the responses represent patients' intentions. We have analysed a large dataset of 104 patients, which is exceptional for a qualitative study, and the proportions found of patients who thought the interview was an acceptable method gives further credence to this finding.

We were not able to establish if the interview brought distress after the researcher had left. A future study could adopt a longitudinal design and repeated interviews to determine this.

## Conclusion

Most of the participants evaluated the interview as a positive experience and nobody said it had caused distress. This confirms other studies which have focused on the effects of interviews showing an almost general benefit for the participants. Therefore, these positive aspects need to be stressed rather than the risks involved. This could create awareness of their merits to those involved in ethical judgements and facilitate recruitment into such studies. The open interview allows access to those patients or carers who are unable to participate in other study designs and who are therefore usually missed by research. The qualities of such interviews are not only relevant for pure qualitative study purposes but could be integrated into more formalised research designs to promote the personal factor which can be sensitive to patients' needs.

Studies in palliative care need to take an inclusive approach as patients and carers needs are closely linked. We found that they were sometimes reluctant to take part in separate interviews and that it is necessary to consider whether it is acceptable to separate them for the sake of research. Joint and separate interviews can produce completely different findings.

The need for a dynamic consent procedure has been identified before in palliative care research. Here, we encountered problems with written consent, which can be insensitive to the limitations caused by disability and advanced disease. Sometimes we found apprehension towards consent forms, indicating the abandonment of rights, rather than securing them. We need to experiment with flexible and innovative ways of obtaining consent, accommodating the issues that can arise in this population. Retrospective consent (with full information provided at the outset) could offer a solution, allowing participants the time to negotiate consent, and evaluate the interview in retrospect.

## Competing interests

The authors declare that they have no competing interests.

## Authors' contributions

MG conceived the study. IJH contributed substantially to the design of the two studies on which these data are based and to all the procedures of gaining ethics permission. MG and CS conducted the data collection. MG drafted the manuscript. CS and IJH revised it critically for important intellectual content. IJH read and approved the final manuscript.

**Table 1:** Type and involvement of patients and carers

**Types of participants**	**Patients interviewed**	**Age patient**	**Gender patient**	**Settings**	**Type of carer interview**	**Number and relation of carer to patient**	**Gender carer**
Patients with cancer	30	Range: 52–67	8 women	Outpatient clinic	5J	5 spouses	2 men
		Median: 68	22 men		2S	1 daughter	5 women
						1 son	
Patients with COPD	14	Range: 52–75	9 women	Outpatient clinic	2S	5 spouses	6 women
		Median: 69	5 men		4J	1 daughter	
Patients with cardiac failure	10	Range: 61–80	3 women	Outpatient clinic	3S	3 spouses	3 women
		Median: 69	7 men				
MND patients	10	Range: 24–77	1 woman	Outpatient clinic	9S	8 spouses	9 women
		Median: 42	9 men			1 mother	
Patients with severe COPD	4	Range: 52–78	2 women	Community	1J	2 spouses	1 man
		Median: 70	2 men				
Patients needing palliative care	8	Range: 64–79	5 women	Community	1S	1 spouse	2 women
		Median: 69	3 men		1J	1 daughter	

J: joint, S: separate

## Pre-publication history

The pre-publication history for this paper can be accessed here:



## Supplementary Material

Additional file 1 Type and involvement of patients and carers. The data provided shows the conditions, age and gender of patients, where they were recruited, their relationship with the carer, their age, and whether the carer's interview was separate from or conducted jointly with the patient Click here for file
