# Supplementary material for: Is the qualitative research interview an acceptable medium for research with palliative care patients and carers?
Source: BMC Med Ethics. 2008 Apr 24;9:7. doi: 10.1186/1472-6939-9-7 (PMC2383914; doi:10.1186/1472-6939-9-7)
Supplement: Additional file 1 — Type and involvement of patients and carers. The data provided shows the conditions, age and gender of patients, where they were recruited, their relationship with the carer, their age, and whether the carer's interview was separate from or conducted jointly with the patient [file 1472-6939-9-7-S1.doc]

**Table 1: Type and involvement of patients and carers**

| **Types of participants** | **Patients interviewed** | **Age patient** | **Gender patient** | **Settings** | **Type of carer interview** | **Number and relation of carer to patient** | **Gender carer** |
| --- | --- | --- | --- | --- | --- | --- | --- |
| Patients with cancer | 30 | Range:  52-67  Median:  68 | 8 women  22 men | Outpatient clinic | 5J  2S | 5 spouses  1 daughter  1 son | 2 men  5 women |
| Patients with COPD | 14 | Range:  52-75  Median:  69 | 9 women  5 men |  | 2S/4J | 5 spouses  1daughter | 6 women |
| Patients with cardiac failure | 10 | Range: 61-80  Median: 69 | 3 women  7 men |  | 3S | 3 spouses | 3 women |
| MND patients | 10 | Range: 24-77  Median: 42 | 1 woman  9 men |  | 9S | 8 spouses  1 mother | 9 women |
| Patients with severe COPD | 4 | Range: 52-78  Median: 70 | 2 women  2 men | Community | 1J | 2 spouses | 1 man |
| Patients needing palliative care | 8 | Range: 64-79  Median: 69 | 5 women  3 men |  | 1S/1J | 1spouse  1daughter | 2 women |

J: joint, S: separate
